# Huntingtin Aggregates in the Olfactory Bulb in Huntington’s Disease

**DOI:** 10.3389/fnagi.2020.00261

**Published:** 2020-08-18

**Authors:** Blake Highet, Birger Victor Dieriks, Helen C. Murray, Richard L. M. Faull, Maurice A. Curtis

**Affiliations:** ^1^Department of Anatomy and Medical Imaging, Faculty of Medical and Health Science, The University of Auckland, Auckland, New Zealand; ^2^Centre for Brain Research, Faculty of Medical and Health Science, The University of Auckland, Auckland, New Zealand

**Keywords:** Huntington’s disease, olfactory bulb, anterior olfactory nucleus, huntingtin aggregates, tau

## Abstract

Olfactory deficits are an early and prevalent non-motor symptom of Huntington’s disease (HD). In other neurodegenerative diseases where olfactory deficits occur, such as Alzheimer’s disease and Parkinson’s disease, pathological protein aggregates (tau, β-amyloid, α-synuclein) accumulate in the anterior olfactory nucleus (AON) of the olfactory bulb (OFB). Therefore, in this study we determined whether aggregates are also present in HD OFBs; 13 HD and five normal human OFBs were stained for mutant huntingtin (mHtt), tau, β-amyloid, TDP-43, and α-synuclein. Our results show that mHtt aggregates detected with 1F8 antibody are present within all HD OFBs, and mHtt aggregate load in the OFB does not correlate with Vonsattel grading scores. The majority of the aggregates were located in the AON and in similar abundance in each anatomical segment of the AON. No mHtt aggregates were found in controls; 31% of HD cases also contained tau neurofibrillary tangles within the AON. This work demonstrates HD pathology in the OFB and indicates that disease-specific protein aggregation in the AON is a common feature of neurodegenerative diseases that show olfactory deficits.

## Introduction

Huntington’s disease (HD) is an autosomal, dominantly inherited neurodegenerative condition characterized by abnormalities in movement, psychiatric disturbances, and cognitive deterioration (Thompson et al., 1988). In HD, an expansion in the trinucleotide CAG repeat present in the first exon of the HTT gene results in the production of mutant huntingtin protein (mHtt) with an expanded polyglutamine repeat (MacDonald et al., 1993). Individuals who carry more than 37 CAG repeats in one of their two HTT alleles are at a high risk of developing HD during their lifetime, with the CAG tract length inversely correlated with the age of onset (Duyao et al., 1993).

In addition to the well-characterized movement and cognitive deficits, several studies also report impairments in olfaction. A small retrospective study showed marked deficits in odor recognition in pre-clinical HD patients (Moberg et al., 1987). Further studies have shown that odor recognition is not affected (Nordin et al., 1995) but that pre-clinical impairments include compromised odor memory and identification (Bylsma et al., 1997; Hamilton et al., 1999; Pirogovsky et al., 2007), as well as impaired strength and quality discrimination (Nordin et al., 1995). Some studies have identified odor detection deficits (Nordin et al., 1995; Moberg and Doty, 1997), while others have identified recognition deficits in symptomatic HD patients (Lazic et al., 2007). These findings suggest that the olfactory deficits are most likely arising from more than one part of the olfactory system.

In this study, we investigated a potential physical basis for olfactory impairments by examining olfactory bulbs (OFBs) for the presence of mHtt. Oligomeric mHtt can reduce endoplasmic reticulum (ER)-associated protein degradation and thereby induce both mitochondrial and ER stress responses that contribute to cell dysfunction (Leitman et al., 2013; Kumar et al., 2014). The formation of aggregated mHtt is considered to be a downstream consequence of oligomerization that sequesters these toxic oligomers, effectively buffering their effect on ER stress (Leitman et al., 2013). Therefore, the presence of mHtt aggregates is an indication that the cell is attempting to mitigate the ER stress effects of toxic oligomers (Leitman et al., 2013). We hypothesize that mHtt aggregates in the OFB would indicate cellular dysfunction in this brain region that could contribute to olfactory symptoms.

Recent evidence indicates that there is temporal relationship between aggregate formation in the bulb and olfactory symptoms in several neurodegenerative diseases. In Alzheimer’s disease and Parkinson’s disease, accumulation of β-amyloid, tau, and α-synuclein respectively, occurs in the OFB early in the disease when olfactory symptoms are present (Kovács et al., 2001; Braak et al., 2003; Zapiec et al., 2017). Furthermore, transgenic HD mice show olfactory deficits, and huntingtin aggregates appear early in the OFB and olfactory tubercle (Menalled et al., 2003; Hölter et al., 2013). Unfortunately, very little data in rodents are available that combine quantification of mHtt aggregates with olfactory testing. In Alzheimer’s disease and Parkinson’s disease, aggregates primarily accumulate in a subregion of the OFB called the anterior olfactory nucleus (AON). The AON is the neural conduit between the OFB and the piriform cortex, entorhinal cortex, amygdala and hippocampal formations and is involved in secondary olfactory processing (Shepherd, 1972; Ubeda-Bañon et al., 2010; Mason et al., 2016). The AON is delineated into bulbar (AONb), interpeduncular (AONi), retrobulbar (AONr), and cortical (AONc) divisions depending on their position within the olfactory bulb or tract (Ubeda-Bañon et al., 2010). However, it remains unclear whether these substructures are separate or connected throughout the OFB and tract in humans. As the AON is specifically affected by aggregates in several diseases, it is hypothesized to be an area sensitive to cell stress.

In this study, we investigated whether the HD brain has aggregate pathology in the OFB, with particular focus on the AON. In addition to the screening for mHtt aggregates, other disease-associated protein aggregates were also assayed including β-amyloid plaques, tau neurofibrillary tangles, phospho TDP-43 aggregates, and α-synuclein Lewy bodies and fibrils, providing a complete pathological picture of the OFB in HD. Evidence of mHtt in the OFB may partially explain the olfactory deficits observed in both preclinical and clinical HD.

## Materials and Methods

### Human Brain Tissue

The post-mortem adult human brain tissue for this study was obtained from the Neurological Foundation Human Brain Bank (Centre for Brain Research, The University of Auckland). The University of Auckland Human Participants Ethics Committee approved the protocols used in these studies (approval# 011654). Written, informed consent was obtained from the individual(s) and/or next of kin to use the tissue and for the publication of any potentially identifiable data included in this article.

Olfactory bulbs from 13 HD cases and 5 neurologically normal cases with no history of neurological disease were collected and used in this study. The HD cases had an average age of 60.2 years (range 29–80 years), with a post-mortem interval between 2 and 25 h after death (average post-mortem interval 15.5 h) and included 3 females and 10 males. HC128 is heterozygous with CAG repeat length >40 as well as a normal repeat. The normal cases had an average age of 64.4 years (range 57–85 years), with a post-mortem interval between 20 and 56 h after death (average post-mortem interval 32.8 h) and included 2 females and 3 males (Table 1).

**TABLE 1:** Brain tissue used in this study.

Case number	Age (years)	Sex	Post-mortem delay (hours)	Cause of death	Vonsattel grade	CAG repeat length	Additional pathology in olfactory bulb
HC123	54	M	14	Bronchopneumonia	HD-4	23/47	–
HC128	80	M	13	Myocardial infarct	HD-3	>40	β-amyloid plaques and tau staining throughout bulb
HC131	70	M	2	Unknown	HD-3	19/45	–
HC148	63	M	16	Pulmonary embolism	HD-3	22/43	–
HC150	64	F	21	Bronchopneumonia	HD-2	22/42	–
HC152	60	M	16	Kidney failure	HD-3	23/43	–
HC153	70	M	–	Unknown	HD-3	17/42	–
HC155	73	F	6	Respiratory arrest	HD-4	30/47	–
HC156	47	M	16	Upper airway obstruction	HD-2	17/42	Tau staining throughout bulb
HC158	52	F	19	Severe progressive HD	HD-2	23/45	–
HC159	29	M	19	Systemic inflammatory disease secondary to burning	HD-4	12/58	–
HC163	59	M	19	Unknown	HD-3	15/42	Tau staining in olfactory tract
HC164	62	M	25	Ischemic heart disease	HD-2	16/43	Tau staining throughout bulb
H230	57	F	32	Carcinomatosis	Normal	Not determined	–
H242	61	M	20	Coronary atherosclerosis	Normal	Not determined	–
OFB51	85	M	20	Carbon dioxide poisoning	Normal	Not determined	Some β-amyloid plaques and tau staining throughout bulb
OFB55	56	M	56	Myocardial infarct	Normal	Not determined	Small amount of phosphorylated alpha synuclein
OFB57	63	F	36	Presumed diabetes	Normal	Not determined	–

For the immunohistochemical studies, the human brains were processed as previously described (Waldvogel et al., 2006). Upon arrival of the brain to the Brain Bank, the OFBs were removed from the rest of the brain and immersed in 15% formalin in 0.1M phosphate buffer for a minimum of 24 h at room temperature. Before embedding in paraffin, OFBs were dehydrated in a series of progressive ethanol baths (70%, 80%, 2 × 95% and 2 × 100% for 30 min each), before being cleared in chloroform (2 × 30 min) followed by paraffin infiltration. This was an automated process completed in a tissue processor (Leica BioSystems); 7-μm-thick sections from paraffin embedded olfactory bulb blocks were cut using a rotary microtome (Leica Biosystems RM2235) and mounted onto Superfrost-Plus charged slides (Menzel-Glaser) in a water bath set to 44°C (Leica Biosystems H1210). Sections were left to dry for 72 h at room temperature prior to immunohistochemical staining.

### Immunohistochemistry Procedures

Tissue was processed as described previously (Dieriks et al., 2018). Sections were heated for 1 h at 60°C on a heating block, dewaxed in xylene (2 × 30 min) and rehydrated through an alcohol series (100%, 95%, 80%, 75%). Antigen retrieval was performed by incubating the slides in a pressure cooker in Tris-EDTA buffer pH 9.0 and left to cool down in the cooker for 1.5 h. When staining for 1C2, sections were additionally treated with 80% formic acid at RT for 3 min, followed by extensive washing. Paraffin sections were permeabilized in PBS containing 0.1% Triton X-100 at 4°C for 10 min. Sections were then blocked in 10% normal goat serum in PBS for 1 h at RT. Subsequently, the sections were incubated with primary antibodies overnight at 4°C (Supplementary Table S1). Sections were then incubated with the corresponding goat secondary Alexa Fluor (488, 594, 647) conjugated secondary antibody (Invitrogen) or avidin-Alexa Fluor 647 at 1:500 for 3 h at RT. For R-Kif5A specifically 1× universal immuno-peroxidase polymer anti-rabbit (414142F, Nichirei Biosciences Inc.) was applied for 3 h in 1% NGS, followed by PBS washes 3× for 5 min. Sections were then incubated with a Tyramide SuperBoost^TM^ Kit (Invitrogen B40922; 0.2× Alexa Fluor^TM^ 488 tyramide reagent, 0.03% H_2_O_2_ in 1× reaction buffer) for 15 min at 37°C, followed by PBS washes 3× for 10 min. Finally, sections were incubated with Hoechst 33342 (Invitrogen H3570) at 1:20,000 for 5 min at RT and then cover slipped with ProLong diamond antifade reagent (Invitrogen P36965). All washes were done with PBS (3× for 5 min). Control sections where the primary antibody was omitted showed no immunoreactivity. The control experiments showed that the secondary antibodies had no cross-reactivity. Fluorescent images were captured with a MetaSystems VSlide slide scanner with a 20× dry lens (NA 0.9). Confocal images were acquired using a FV1000 confocal microscope (Olympus, Japan) with a 40× magnification oil immersion lens (1.00 NA), 60× magnification oil immersion lens (1.35 NA) or 100× magnification oil immersion lens (1.40 NA).

### Quantification

Three OFB sections 500 μm apart were selected per case for this study. The first section chosen was a mid-sagittal section through the OFB. The other two sections were then chosen 500 μm on either side of the first mid-sagittal section. Sections stained for 1F8 were used for the quantification of mHtt aggregates. The images were extracted from VSViewer, and the AONs were segmented from each OFB on ImageJ using the polygon selection tool. The AON regions were determined based on a combination of staining patterns. Namely, PGP9.5 staining, which labels the AON compartment and Hoechst staining that has less signal abundance in the AON due to the presence of large diffusely stained neuronal nuclei (Gardner et al., 2017; Stevenson et al., 2020). Following the segmentation, a precise measurement of the AON area was made. To obtain a background staining intensity measure for the 1F8 staining, a 50 μm × 50 μm square (area = 2500 μm^2^) was placed over three different areas of background staining, and the grayscale pixel value was measured. The background measurements were averaged to give a mean background staining intensity value. The multipoint tool was used to determine the number of 1F8^+^ cells. To be counted, a cell needed to have a 1F8^+^ mHtt aggregate that was within or very close to the nucleus. If this criterion was satisfied, then the single largest aggregate within/close to the nucleus was selected with the multipoint tool. Once all the apparent aggregates had been selected, the grayscale pixel value was measured for every selection point. To be considered a true aggregate, the grayscale pixel value for each selection point had to be higher than a pre-determined threshold (45 grayscale points). To obtain total 1F8^+^ mHtt cell density values (cells/mm^2^), the total number of 1F8^+^ mHtt cells counted across the three sections for each case was then divided by the total measured cross-sectional area of the AON in those three sections. Counts were done by two individuals (BH and BVD) who were blinded to the case number, disease status and Vonsattel grade.

### Statistical Analysis

In general, data are presented as mean ± standard deviation (SD) from the average of three different sections per case. Data visualization and statistical hypothesis testing were performed using GraphPad Prism^®^ Version 8.02. Linear regression was used to analyze correlations. One-way analysis of variance (ANOVA) was used when comparing across cell types with Tukey’s multiple comparison adjustment. Repeated-measures ANOVA was used for assessment of 1F8^+^ mHtt cell density across AON substructures within a case. Statistical significance was set as *p* < 0.05.

## Results

### Huntingtin (mHtt) Aggregates Are Primarily Found Within the AON of Human OFBs

**FIGURE 1:**
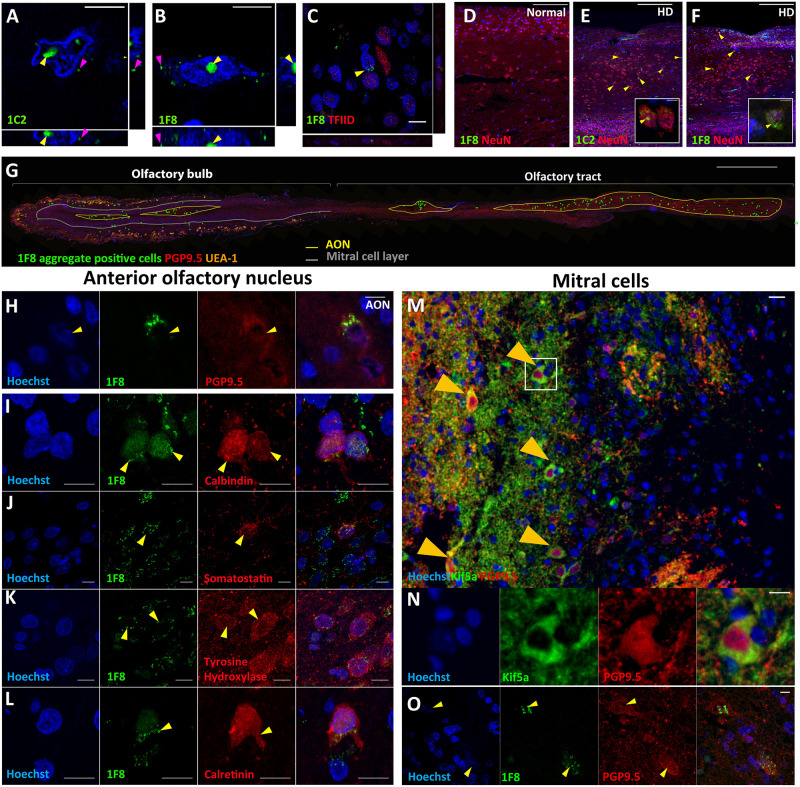
Fluorescent staining for Huntingtin (Htt) aggregates in human olfactory bulbs (OFBs). Confocal imaging of nuclear (yellow arrows) and cytoplasmic (magenta arrows) mHtt aggregates identified with 1F8 and 1C2 antibodies **(A,B)**. No overlap is seen between 1F8 and TFIID, indicating 1F8 solely detects mHtt aggregates **(C)**. Cells with 1F8^+^ and 1C2^+^ aggregates were only found in olfactory bubs from cases with Huntington’s Disease (HD). No 1F8^+^ aggregates were found in normals **(D)**. 1C2 and 1F8 staining were done on sequential sections that detect similar amounts of cells with mHtt aggregates (yellow arrows). The inserts show representative images of NeuN^+^ cells with mHtt aggregates **(E,F)**. Distribution of 1F8^+^ cells (green dots) throughout a sagittal olfactory bulb section. Majority of cells are found within the anterior olfactory nucleus (AON) **(G)**. 1F8^+^ mHtt aggregates (yellow arrows) are found in PGP9.5^+^neurons within the AON **(H)**. Double labelling of 1F8^+^ mHtt cells in the anterior olfactory nucleus with neuronal markers. Yellow arrows indicate that 1F8^+^ mHtt aggregates are found in AON neurons that stain for calbindin **(I)**, somatostatin **(J)**, tyrosine hydroxylase **(K)** and calretinin **(L)**. Mitral cells (orange arrows) co-express PGP9.5 and Kif5a **(M,N)**. 1F8^+^ mHtt aggregates were found in mitral cells **(O)**. Scale bars represent 10 μm except for **(D–G)** 500 μm and M 20 μm.

To detect mHtt aggregates in the olfactory bulb of HD cases, we tested two commonly used antibodies, 1C2 and 1F8. Both antibodies detect aggregated polyglutamine (polyQ) repeat structures of length above 38 and have been used previously to detect mHtt aggregates (Trettel et al., 2000). 1C2^+^ and 1F8^+^ aggregates were found in the nucleus and cytoplasm, with the majority of cells containing nuclear aggregates (Figures 1A,B). We also tested whether the staining we observed could be due to TATA binding protein (TBP) rather than mHtt aggregates. The 1C2 and 1F8 antibodies were raised against TBP which consists of a polyQ segment between 25 and 42 repeats long (Herndon et al., 2009). We found no overlapping staining between the 1F8 antibody and the TFIID antibody that specifically detects TBP (Figure 1C). No 1F8^+^ mHtt aggregates were found within any normal OFBs (Figure 1D). The density of cells bearing 1F8^+^ or 1C2^+^ aggregates identified in neuronal nuclei (NeuN)^+^ cells on adjacent sections across the entire OFB were similar (11.5 vs. 11.0 and 3.7 vs. 3.6 cells/mm^2^) (Figures 1D–F). Given there were no differences in cell density counts and the 1F8 antibody does not require formic acid antigen retrieval, which impaired detection of other cellular markers, we performed the subsequent quantification of mHtt aggregates using the 1F8 antibody.

In HD OFBs, 1F8^+^ mHtt aggregates were found in the glomerular layer, external plexiform, mitral cell layer, internal plexiform layer, granule cell layer, and in multiple AON compartments throughout the olfactory tract. The majority of 1F8^+^ mHtt aggregates were observed within the AON (Figure 1G). To confidently identify the AON regions across different sections and cases, we found that co-labeling with Hoechst and PGP9.5 was sufficient. The AON has an increased PGP9.5 immunoreactivity and a reduced number of Hoechst positive cells that have large diffuse nuclei compared to the neighboring layers. As a result, the AON has lower overall Hoechst staining (Figure 1G).

To determine whether the 1F8^+^ aggregates specifically accumulated within certain subsets of AON neurons, we investigated co-labeling of 1F8^+^ mHtt aggregates with various neuronal markers. We found 1F8^+^ mHtt aggregates within calbindin^+^, somatostatin^+^, tyrosine hydroxylase^+^, and calretinin^+^ AON cells (Figures 1I–L). This indicates a wide variety of AON neuron populations contain 1F8^+^ mHtt aggregates.

In addition, 1F8^+^ mHtt aggregates were found in the nucleus and cytoplasm of cells that resembled mitral cells with large diffuse nuclei that stain less intensely with Hoechst and label with PGP9.5 and the putative mitral cell marker Kif5A (Kanai et al., 2000) (Figures 1M–O).

### Quantification of mHtt Aggregates in the AON

**FIGURE 2:**
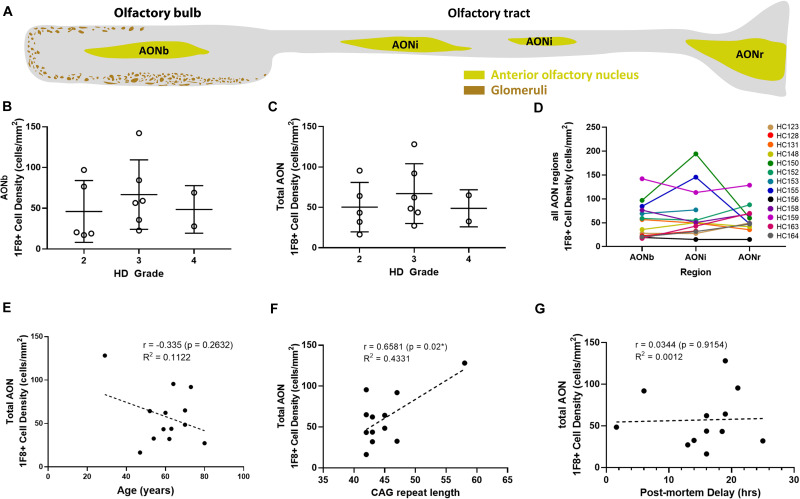
Schematic overview of human olfactory bulb and 1F8^+^ mHtt cell density counts. Representative diagram of a sagittal human olfactory bulb sections indicating the location of the bulbar (AONb), interpeduncular (AONi), and retrobulbar AON (AONr) **(A)**. 1F8^+^ mHtt cell density across Vonsattel grades for the AONb **(B)** and all AON regions combined **(C)**. 1F8^+^ mHtt cell density across the AON segments for each HD case **(D)**. Correlations between 1F8+ mHtt cell density for the total AON and age, CAG repeat length, and post-mortem delay (PMD) **(E–G)**.

Most cells with 1F8^+^ mHtt aggregates were found within the AON. Therefore, we determined 1F8^+^ aggregate density in the AONb, AONi, and AONr substructures separately as well as combined (total AON, Figure 2A) for HD bulbs only (Ubeda-Bañon et al., 2010). In the AONb, 56.4 ± 38.3 cells/mm^2^ contained 1F8^+^ mHtt aggregates versus 58.1 ± 31.8 cells/mm^2^ overall. We did not detect any significant difference in 1F8^+^ mHtt cell density across HD cases grouped by Vonsattel grading (AONb, *p* = 0.813; AON total, *p* = 0.812; Figures 2B,C). Within each case, we also did not detect any gradient in 1F8^+^ mHtt cell density across the AON substructures from bulb to tract (Figure 2D). No correlation was observed between age at death and AONb 1F8^+^ mHtt cell density (*r* = -0.3891, *p* = 0.1889, *R*^2^ = 0.1514, graph not shown) and total AON 1F8^+^ mHtt cell density (*r* = -0.335, *p* = 0.2632, *R*^2^ = 0.1122, Figure 2E). A positive correlation was observed between CAG repeat length and AONb 1F8^+^ mHtt cell density (*r* = 0.687, *p* = 0.0136^∗^, *R*^2^ = 0.4719, graph not shown) and total AON 1F8^+^ mHtt cell density (*r* = 0.6581, *p* = 0.02^∗^, *R*^2^ = 0.4331, Figure 2F), with higher cell densities observed in cases with longer CAG repeats. This effect was only seen because the presence of case HC159 having a high CAG repeat. No correlation was observed between post-mortem delay and AONb 1F8^+^ cell density (*r* = -0.01234, *p* = 0.969, *R*^2^ = 0.0002, graph not shown) and total AON 1F8^+^ cell density (*r* = 0.0344, *p* = 0.9154, *R*^2^ = 0.0012, Figure 2G).

### Co-occurrence of Tau Pathology Present in HD Olfactory Bulbs

**FIGURE 3:**
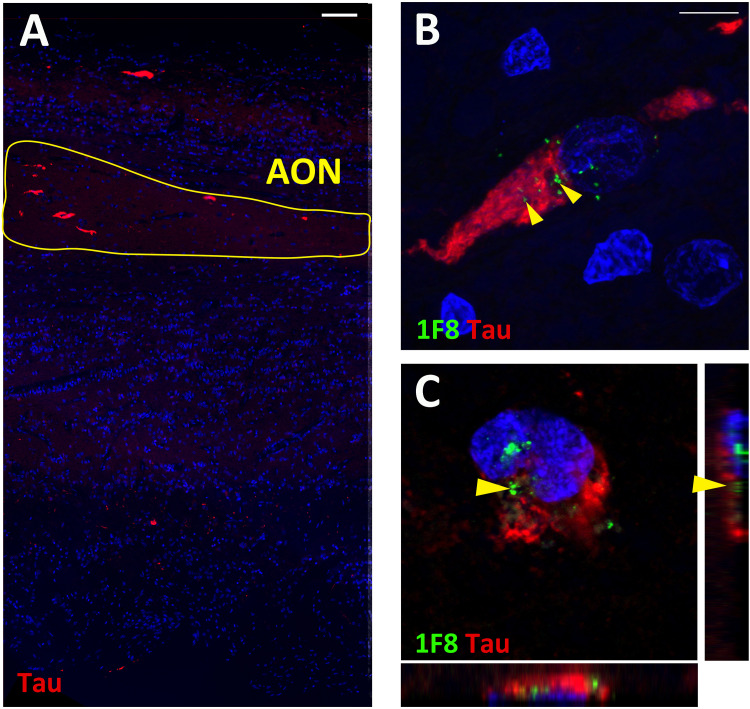
Fluorescent staining for tau and Huntingtin (Htt) aggregates in human olfactory bulbs (OFBs). Overview staining of tau aggregates in the olfactory bulb **(A)**. Representative image of tau (red) and 1F8^+^ mHtt aggregates (green, yellow arrows) in AON neurons **(B)**. Confocal Z-stack projection with orthongal projections of tau and 1F8^+^ mHtt aggregates occupying different subsections of the neurons **(C).** Scale bars represent 100 μm for **(A)** and 10 μm for **(B,C)**.

In addition to 1F8^+^ mHtt aggregates, all OFBs used in this study were screened for the presence of α-synuclein phospho ser129, β-amyloid, tau, and phospho-TDP-43 positive aggregates. No aggregates were found in the five normal cases, except for OFB55 that contained a small amount of α-synuclein in the glomerular layer. OFB51 contained sparse distributed β-amyloid plaques and tau staining throughout the bulb. The levels observed within these normal cases are far below those observed in PD and AD bulbs, respectively (Kovács et al., 2001; Braak et al., 2003; Stevenson et al., 2020). No α-synuclein phospho ser129 or phospho-TDP-43 aggregates were found in the HD OFBs. Diffuse β-amyloid plaques were present in low amounts in HC128, which is normal considering the age of the case (80 years). However, four of the 13 HD cases (31%) contained tau tangles throughout the bulb and tract. No tau was present in the other HD cases and 5 normal cases (Figure 3A). This finding was consistent with pathology reports that found neurofibrillary tangles in the cortex and hippocampus for these cases. Double labeling for tau and 1F8 revealed that a proportion of neurons contain both 1F8^+^ and tau aggregates within the same neuron. Optical sectioning with the confocal microscope revealed that 1F8^+^ mHtt aggregates and tau tangles were present in the same cell but did not overlap (Figures 3A–C). OFB cases that contained neurofibrillary tau tangles had significantly less 1F8^+^ mHtt aggregates in the AON than the cases that do not show any tau immunoreactivity (AONb 19.9 ± 1.2 cells/mm^2^ vs. 73.0 ± 11.5 cells/mm^2^, *p* = 0.0028; total AON 29.83 ± 5.6 cells/mm^2^ vs. 70.3 ± 10.0 cells/mm^2^, *p* = 0.0056).

## Discussion

Olfactory deficits are seen in a range of neurodegenerative disorders; while pathological protein deposition and mechanisms for olfactory deficits have been relatively well explored in both Alzheimer and Parkinson’s disease, there is very limited research into olfactory deficits in HD. This article provides the first description of mHtt aggregates in the HD olfactory bulb, despite deficits in HD olfaction being reported 33 years ago (Moberg et al., 1987).

All HD olfactory bulbs and tracts contained mHtt aggregates. We found more 1F8^+^ mHtt cells in the AON of cases with higher CAG repeat length despite there being no correlation with Vonsattel grade. This correlation with CAG repeat length was driven by one single case (CAG repeat of 58). These observations indicate that the amount of aggregates does not relate to disease severity in the rest of brain, which is logical as Vonsattel grading is largely based on degeneration of the caudate nucleus and does not take into account the abundance of mHtt aggregates. Furthermore, no gradient in 1F8^+^ cell density across AON substructures was observed, suggesting either no detectable progression of aggregate accumulation along the olfactory bulb and tract or that this progression occurs earlier in disease pathogenesis and the AONs are saturated with aggregates by end-stage disease. mHtt aggregates were primarily observed within the AON and mitral cells and were observed within a range of neuronal subtypes. This suggests that the AON is a region susceptible to aggregate formation in HD and potentially plays a role in the olfactory deficits. This is a common feature observed across a range of neurodegenerative diseases but it remains unclear why aggregate pathology is highly concentrated within the AON, whether the disease is sporadic (most AD and PD cases) or purely genetic (HD) (Kovács et al., 2001; Braak et al., 2003; Stevenson et al., 2020).

Studies evaluating mechanisms of neurodegenerative diseases typically focus on the dominant protein pathology in a particular disease, with few studies investigating the frequent comorbidity and interaction of pathological lesions across disorders. In HD, comorbidity is likely underreported as screening for the common protein aggregates (α-synuclein, β-amyloid, tau, TDP-43 and mHtt) is not common practice (Davis et al., 2014). This is despite evidence that protein aggregates can influence the seeding and spread of other aggregates (Bassil et al., 2020). Furthermore, mHtt influences the hyperphosphorylation and localization of tau in an HD mouse model (Blum et al., 2015). Previous studies have suggested that the level of tau phosphorylation may limit the severity and/or progression of HD (McIntosh et al., 1978; Myers et al., 1985; Reyes and Gibbons, 1985; Moss et al., 1988; Caparros-Lefebvre et al., 2009). An in-depth analysis of the available evidence that HD is potentially a tauopathy is reviewed by Gratuze et al. (2016). We explored this further by examining co-occurrence in the bulb. No α synuclein Lewy bodies, β-amyloid plaques (except for HC128), and phospo-TDP-43 were present in the olfactory tract and bulb of the HD cases. In addition to mHtt aggregates, 31% of HD cases contained cells with 1F8^+^ mHtt aggregates and tau tangles. Besides the presence of tau tangles, these cases did not have any other noticeable differences (similar Vonsattel grades, CAG repeat length and ages at death ranging from HD2-3, 42–43 and 47–80 years, respectively). Collectively, these data point toward the idea that mHtt aggregates with or without tau in the OFB play a role in HD olfactory deficits.

Our work provides further evidence that the olfactory bulb is indeed involved in the olfactory deficits observed clinically in HD patients. This suggests a peripheral cause in which dysfunction occurring between the olfactory sensory neurons and the bulb causes alterations in detection (Leopold, 2002). However, this study was limited to brain tissue and did not investigate the olfactory mucosa where the olfactory sensory neurons are located. Therefore, we cannot rule out that alterations in the sensitivity of these neurons and accumulation of mHtt might cause reduced olfactory detection. As highlighted earlier, the absence of data combining mHtt aggregates quantification with olfactory testing limits our understanding of the functional association between these two factors. This currently limits the functional association between mHtt aggregates and olfactory function. Future studies in humans that combine both pathological aggregate staining and patient olfactory history will be critical in defining the association between mHtt aggregates and olfactory function. The accumulation of mHtt in the OFB may contribute to neuronal dysfunction by activating cell stress pathways such as the unfolded protein response (UPR). This endoplasmic reticulum stress response acts to mitigate the accumulation of misfolded proteins. However, chronic UPR activation can lead to apoptosis and the upregulation of beta-amyloid and tau production. UPR activation in relation to tau occurs in the Alzheimer’s disease OFB, which could explain olfactory loss in a subset of our cases (Murray et al., 2020). There is evidence of the UPR activation in HD, but it has not been studied in the OFB (Leitman et al., 2013). Loss of glomeruli may also contribute to olfactory dysfunction, as observed in Parkinson’s disease (Zapiec et al., 2017). This would be an interesting investigation to carry out for HD bulbs. The other olfactory deficits prevalent in HD are central deficits relating to odor identification and discrimination, and these can arise from degeneration or dysfunction in the piriform cortex or other central structures (Nordin et al., 1995; Lazic et al., 2007). From the available literature, it would appear that all levels of the olfactory system are affected in HD, and our work contributes to this literature by providing a possible pathological basis for a peripheral deficit.

## Conclusion

These results show for the first time that mHtt aggregates are present within all the human HD olfactory bulbs in this study and accumulate primarily within the anterior olfactory nucleus. As such, HD parallels similarities with other neurodegenerative disorders where aggregate pathology is largely concentrated in the AON in the OFB.

## Data Availability Statement

The raw data supporting the conclusions of this article will be made available by the authors, without undue reservation.

## Ethics Statement

The studies involving human participants were reviewed and approved by The University of Auckland Human Participants Ethics Committee (approval #011654). Written informed consent was obtained from the individual(s) and/or next of kin as part of the brain donation process.

## Author Contributions

BH performed the experiments and analyzed the data. BD designed the study, performed the experiments, and analyzed the data. HM analyzed the data. RF and MC organized the collection of the human brain tissue and directed the work. BH, BD, HM, RF, and MC wrote the manuscript. All authors contributed to the article and approved the submitted version.

## Conflict of Interest

The authors declare that the research was conducted in the absence of any commercial or financial relationships that could be construed as a potential conflict of interest.
