# Supplementary material for: Huntingtin Aggregates in the Olfactory Bulb in Huntington’s Disease
Source: Front Aging Neurosci. 2020 Aug 18;12:261. doi: 10.3389/fnagi.2020.00261 (PMC7461834; doi:10.3389/fnagi.2020.00261)
Supplement: Supplementary file 1 [file Table_1.DOCX]

Suppl. Table 1: Primary antibodies and lectin used in this study. These primary antibodies have been extensively validated on human brain sections in our group and/or are included in the Journal of Comparative Neurology (JCN) antibody database. The following mouse (M), and rabbit (R) antibodies were used:

| Antigen | Dilution | Catalog # | Company/Source | Validation | |
| --- | --- | --- | --- | --- | --- |
| M-1F8 | 1:2000 | 2006 | Marcy Macdonald | (Owada, 2008) |  |
| M-1C2 | 1:1000 | MAB1574 | Millipore | (Herndon et al., 2009)[JCN] |  |
| R-TFIID | 1:1000 | sc-273 | Santa Cruz |  |  |
| R-PGP9.5 | 1:1000 | Ab8189 | Abcam | (Stevenson et al., 2020) |  |
| R-Calretinin | 1:3000 | CR7697 | Swant | (Murray et al., 2016) [JCN] |  |
| R-Calbindin | 1:2000 | CB38a-RF368 | Swant | (Murray et al., 2016)[JCN] |  |
| R-Tyrosine Hydroxylase | 1:1000 | Ab76442 | Abcam | (Zapiec et al., 2017) |  |
| R-Somatostatin | 1:2500 | SAB4502861 | Sigma | [JCN] |  |
| R-Kif5a | 1:150 | HPA004469 | Sigma |  |  |
| R-Tau | 1:1000 | A0024 | Dako | (Murray et al., 2016) |  |
| R-α-synuclein (phospho S129) | 1:3000 | Ab52168 | Abcam | (Stevenson et al., 2020) |  |
| M-β-amyloid (IgG) | 1:100 | M0872 | Dako | (Murray et al., 2016) |  |
| Ulex Europaeus Agglutinin I-biotin (UEA-1) | 1:500 | 046M4129V | Sigma | (Stevenson et al., 2020) |  |
